# A Comparison of Methods for RNA-Seq Differential Expression Analysis and a New Empirical Bayes Approach

**DOI:** 10.3390/bios3030238

**Published:** 2013-06-28

**Authors:** Sergiusz Wesolowski, Marc R. Birtwistle, Grzegorz A. Rempala

**Affiliations:** 1 Department of Mathematics, Florida State University, Tallahassee, FL 32306, USA; E-Mail: wesserg@gmail.com; 2 Department of Pharmacology and Systems Therapeutics, Mount Sinai School of Medicine, New York, NY 10029, USA; E-Mail: marc.birtwistle@mssm.edu; 3 Division of Biostatistics, The Ohio State University, Columbus, OH 43210, USA

**Keywords:** next-generation sequencing, empirical Bayes, gene expression data

## Abstract

Transcriptome-based biosensors are expected to have a large impact on the future of biotechnology. However, a central aspect of transcriptomics is differential expression analysis, where, currently, deep RNA sequencing (RNA-seq) has the potential to replace the microarray as the standard assay for RNA quantification. Our contributions here to RNA-seq differential expression analysis are two-fold. First, given the high cost of an RNA-seq run, biological replicates are rare, and therefore, information sharing across genes to obtain variance estimates is crucial. To handle such information sharing in a rigorous manner, we propose an hierarchical, empirical Bayes approach (R-EBSeq) that combines the Cufflinks model for generating relative transcript abundance measurements, known as FPKM (fragments per kilobase of transcript length per million mapped reads) with the EBArrays framework, which was previously developed for empirical Bayes analysis of microarray data. A desirable feature of R-EBSeq is easy-to-implement analysis of more than pairwise comparisons, as we illustrate with experimental data. Secondly, we develop the standard RNA-seq test data set, on the level of reads, where 79 transcripts are artificially differentially expressed and, therefore, explicitly known. This test data set allows us to compare the performance, in terms of the true discovery rate, of R-EBSeq to three other widely used RNAseq data analysis packages: Cuffdiff, DEseq and BaySeq. Our analysis indicates that DESeq identifies the first half of the differentially expressed transcripts well, but then is outperformed by Cuffdiff and R-EBSeq. Cuffdiff and R-EBSeq are the two top performers. Thus, R-EBSeq offers good performance, while allowing flexible and rigorous comparison of multiple biological conditions.

## 1. Introduction

Next generation sequencing generally refers to the massively parallel identification of the bases that make up nucleotide sequences and has revolutionized the way we can look at biology. Obtaining the sequence of a human genome once cost on the order of $100,000,000 dollars and took years; now, with next generation sequencing techniques, we obtain such a sequence for on the order of $10,000 to $1,000 in days/weeks [1,2,3,4,5]. This rate of cost decrease is amazingly exceeding that of Moore’s law for semiconductors [5].

**Figure 1:**
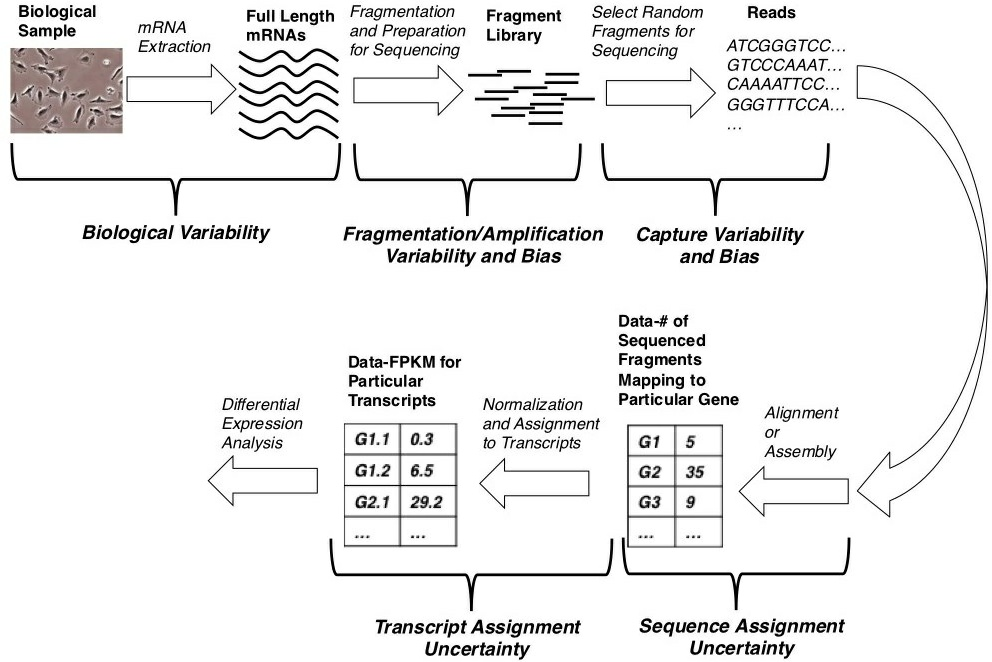
The RNA-seq pipeline. This schematic illustrates the process of going from cells to RNA-seq data, with potential sources of bias and variability noted along the way. See the Introduction for details.

One application of next generation sequencing is transcriptomics, where an entire set of mRNAs is sequenced [6,7]. This application is known as RNA-seq, and the process, from cells to data and differential expression analysis, may be thought of as a pipeline, as shown in Figure 1. Although a wide variety of next generation sequencing platforms exist, they are generally based on slight variations of this core pipeline.

The process, of course, starts with cells, from which mRNA is extracted. Note that, due to the biological variability, the extracted mRNA levels may differ considerably among different biological samples. Then, a so-called library is created from the isolated mRNA. The library typically consists of nucleic acid fragments, but can also consist of full-length nucleic acid molecules [2,8]. It is this library that is the direct input to the next generation sequencer. During library generation, the samples are subject to both fragmentation and amplification variability and bias [2,9]. Because the library generation protocol is sequencing platform-specific, these forms of variability and bias will be platform-dependent (and, even, non-existent in some cases).

A subset of the library molecules is then sequenced, producing what are called reads or DNA sequences. Because only a subset of the library molecules is chosen for sequencing, there is variability and potential bias in the selection process [9]. Furthermore, due to the nature of the sequencing process, the identity of each nucleotide in the read is only known in a probabilistic sense, resulting in variability and bias, due to base-calling errors [2].

With this sample of reads, one can either align them to a reference genome or perform a *de novo* assembly and, subsequently, count the number of reads that align to particular genes. Such aligned reads are also termed mapped reads. Regardless of which of these two are done (alignment or assembly); however, there will inevitably be uncertainty in assigning a read to a particular gene; a single read may well align to multiple genes.

Some researchers consider these count data to be the final product of an RNA-seq experiment. However, such count data are not usually linearly proportional to the original number of full length transcripts for two main reasons. First, longer transcripts generate more reads simply due to their length. Second, because a single gene typically codes for multiple transcripts with different lengths, a gene’s expression in terms of the expected number of counts may remain constant even while the number of transcripts being produced from that gene changes. Moreover, since one eventually wants to compare the results of two or more different sequencing runs, it is also necessary to correct for the total sequencing depth, which is related to how large the subset of the sequenced library molecules is. These reasons are why many people take an additional step to transform these gene counts to units of reads (R) or fragments (F) per kilobase of transcript length per million mapped reads (RPKM or FPKM), which are linearly proportional to original transcript levels [6,7].

Finally, one can assign an FPKM value to each individual transcript for every gene. This transcript assignment process also introduces uncertainty, because only reads that happen to contain exons or exon-exon boundaries that are unique to particular transcripts can inform this transcript assignment process and such reads may be rare [7]. It is with these FPKM data for individual transcripts that we would like to perform differential expression analysis.

In what follows, we first analyze the RNA-seq process described by Figure 1 in a statistical sense, to determine how it may be best modeled. Next, we propose a new empirical Bayes framework for differential expression testing, which uses the well-known and established Cufflinks software [7,9] to generate FPKM measurements, and, subsequently, couples to a modified version of the EBArrays framework, which was originally developed for microarray data analysis [10,11]. The advantages of this so-called R-EBSeq approach are (1) a rigorous treatment of information sharing across genes, which allows for better variance estimates, given the fact that one rarely has biological replicates, due to cost considerations; and (2) the ability to do simultaneous differential expression comparisons with arbitrary many expression patterns. Unlike another EBArrays-based method for the RNA-seq analysis [12], ours works with FPKM measurements, which seem more appropriate for the across-gene information-sharing framework of EBArrays. To illustrate the advantages of our approach, we compare R-EBSeq method with three other established differential expression methods: FPKM-based Cuffdiff and count-based DESeq and BaySeq [7,9,13,14].

## 2. Methods

### 2.1. EBarrays

The method developed in [10,11] characterizes the distribution of expression measurements for a single gene (or transcript) in a certain condition. Here, we present a brief overlook of the idea behind the framework and how we adapt it to our problem. Similar empirical-based approach, but for count-based RNA-seq analysis, has been recently developed in [12]. We refer to their software documentation for additional EBArrays model details.

Let *j* indicate *j*-th transcript and **x**_j_ = *x_j_*_1_,...,*x_jI_* be the expressions in the particular condition denoted: *x*, where *I* is the number of expression measurements we can take in this condition. The method treats **x_j_** as a random sample with a transcript- and condition-specific mean. Thus, we have that *X_ij_|μ_j_* are iid with a pdf given as *f*_*X*_*ij*_*|μ*_*j*__ (*x_ij_*). To allow information sharing among all transcripts and conditions, a prior distribution, *π*, is assumed on *μ_j_*.

In a simple setting, if we investigate differences in expression between only two conditions, the index set {1,...,*I*} is partitioned into two subsets, *s*_1_*,s*_2_, each containing indices for a corresponding condition (**x***_j_* = (**x**_*js*_1__, **x**_*js*_2__)). For each transcript, we aim to investigate a null hypothesis that assumes equivalent expression (*EE_j_*) among conditions, against the alternative that a transcript is differentially expressed (*DE_j_*) in one of conditions.

The pdf in each expression pattern (’EE’ or ’DE’) can be written as follows:

*EE_j_*:

(1) $$f_{0}\left(x_{j}\right)=\int \left(\prod_{i=1}^{I}f_{X_{j}}|\mu_{j}\left(x_{ji}\right)\pi \left(\mu_{j}\right)\right)d\mu_{j}$$

*DE_j_*:

(2) $$f_{1}\left(x_{j}\right)=f_{0}\left(x_{js_{1}}\right)f_{0}\left(x_{js_{2}}\right)$$

Let *p* and 1 *− p* denote the fraction of all genes that are differentially expressed and equivalently expressed among conditions. Then, the pdf for **x***_j_* takes the following form:

(3) $$f_{X}\left(x_{j}\right)=\left(1-p\right)f_{0}\left(x_{j}\right)+pf_{1}\left(x_{j}\right)$$

Using Bayes’ formula, we can write the pdfs for each hypothesis:

(4) $$P\left(H_{{\mathrm{DE}}_{j}}|x_{j}\right)=\frac{P\left(x_{j}|H_{DE_{j}}\right)}{f\left(x_{j}\right)}=\frac{pf_{1}\left(x_{j}\right)}{\left(1-p\right)f_{0}\left(x_{j}\right)+pf_{1}\left(x_{j}\right)}$$

(5) $$P\left(H_{EE_{j}}|x_{j}\right)=\frac{\left(1-p\right)f_{0}\left(x_{j}\right)}{\left(1-p\right)f_{0}\left(x_{j}\right)+pf_{1}\left(x_{j}\right)}$$

The form of both the conditional distribution of *x_j_* and its conjugate prior to the mean are usually known. EBarrays uses the EM algorithm to compute the estimates of parameters for both distributions, as well as mixing proportions of genes supporting the investigated hypothesis.

This method is easily generalized for more than two expression patterns. A pattern corresponding to the null hypothesis, where each sample has the same underlying mean, is always distinguished. Consider *m* DE patterns, which yield a total of *m*+1 patterns (including the null pattern—Equivalent Expression).

The formula for the marginal distribution of **x_j_**, corresponding to Equation (3), is:

(6) $$f_{X_{j}}\left(x_{j}\right)=\sum_{k=0}^{m}p_{k}f_{k}\left(x_{j}\right)$$

where *f_k_* is density corresponding to the *k*-th pattern, as in [10], and *p_k_* is the fraction of genes supporting the corresponding pattern (hypothesis). Consequently, the posterior probability of expression pattern, *k*, for gene, *j*, is:

(7) $$P\left(H_{kj}|x_{j}\right)=\frac{p_{k}f_{k}\left(x_{j}\right)}{f_{X_{j}}\left(x_{j}\right)}$$

### 2.2. EBarrays Model for Cufflinks-Processed Data

Usually, Cufflinks-processed data does not have replicates. To use the EBarray framework, replicates need to be generated from the Cufflinks-specified normal distribution [7] for gene- or transcript-specific FPKM values (see below for discussion of normal approximation). They are then fed into EBarrays. The number of replicates to use is a free parameter of R-EBSeq, and its effect of differential expression analysis is explored in the main text. Since expression measurements approximately follow a normal distribution, the conjugate prior distribution for means is also normal. Thus, it is natural to assume a normal-normal model for the EBarrays estimation procedure:

(8) $$X_{j}|\mu_{j}∼N\left(\mu_{j},\sigma_{j}^{2}\right),\mu_{j}∼N\left(\mu_{0},\tau_{0}^{2}\right)$$

In this setting, *π* ∼ *N*(*μ*_0_*,τ*_0_) and an *n*-dimensional input for gene *j*, after evaluating the integral as detailed in [11], follows a marginal pdf, *f*, which is the pdf of a normal distribution with mean, *μ* = (*μ*_0_,...,*μ*_0_), and covariance matrix:

(9) $$\Sigma_{j}=\sigma_{j}^{2}\times {𝕀}_{n\times n}+\tau_{0}\times M_{n\times n}$$

where 𝕀*_n×n_* is an identity matrix and *M_n×n_* is a matrix of ones.

We adapted the EBarrays R code to account for the normal-normal model with modified variances by modifying the previously built-in LNNMV model (available as Supplemental File). This normal-normal model with modified variances code was created using the tools provided in EBarrays R package [10,11].

### 2.3. Justification for a Normal Distribution Model for Transcript Abundances A_t_

According to Equation (15), the transcript abundance, *Â_t_*, is a product of two maximal likelihood estimates and is approximated in the model by a Gaussian variate. Here, we derive a brief justification, which works for any products of asymptotically Gaussian estimators. Consider two parameters, *θ* and *η*, with their respective estimates, *θ̂_n_* and *η̂_n_*, which satisfy jointly the multivariate CLT, that is, as *n* → *∞*

$$\sqrt{n}\left({\hat{\theta }}_{n}-\theta ,{\hat{\eta }}_{n}-\eta \right)\to BV\;N(0,\sigma_{1},\sigma_{2},\rho )$$

in distribution, where *BV N*(0,*σ*_1_,*σ*_2_,*ρ*) is a bivariate normal random variate with zero marginal means, marginal standard deviations, *σ*_1_ and *σ*_2_, and the correlation coefficient, *ρ*. In the case of the abundance model, *A_t_* considered in the paper, the vector, (*θ̂_n_*, *η̂_n_*) = (*X̂_g_, γ̂_t_*), is a joined MLE and the convergence above follows from the general theory of the likelihood-based estimation (see, e.g., [15] Chapter 4), where, now, *n* stands for the sampling depth (or the overall number of transcripts). Since

$${\hat{\theta }}_{n}{\hat{\eta }}_{n}-\theta \eta ={\hat{\theta }}_{n}{\hat{\eta }}_{n}-\theta \eta -\theta {\hat{\eta }}_{n}+\theta {\hat{\eta }}_{n}={\hat{\eta }}_{n}\left({\hat{\theta }}_{n}-\theta \right)+\theta \left({\hat{\eta }}_{n}-\eta \right)$$

and *η̂_n_* → *η* in probability, therefore, due to Slutsky’s theorem (see, e.g., [15] chapter 1), we see that in distribution,

$$\sqrt{n}\left({\hat{\theta }}_{n}{\hat{\eta }}_{n}-\theta \eta \right)\to N(0,\gamma )$$

where *N*(0,*γ*) is a zero mean normal variate with the standard deviation, *γ*, such that

$$\gamma^{2}=\eta^{2}\sigma_{1}^{2}+\theta^{2}\sigma_{2}^{2}+2\eta \theta \sigma_{1}\sigma_{2}\rho$$

### 2.4. Simulating Data Sets for Use with R-EBSeq

In order to evaluate the performance of our method, we investigated its ROC plots for several different parameter settings under the normal-normal model assumption. The control dataset was created by generating a 1, 000 *× n* measurement matrix, according to the Normal-Normal model—reflecting 1,000 transcripts under one condition with *n* replicates. The number of replicates is also referred to as the sample size.

The treatment dataset was created in the same way, but expression values for 10% of the transcripts (randomly chosen) were generated with altered parameters in order to simulate the differential expression. Only over-expression was considered.

To make the simulations as close to real data as possible, the parameters in the control setting (the mean and the variance) for the generation from the normal-normal model were taken from real data (we have chosen a fixed sample of 1, 000 transcripts from Cufflinks-processed yeast data [16]).

**Figure 2:**
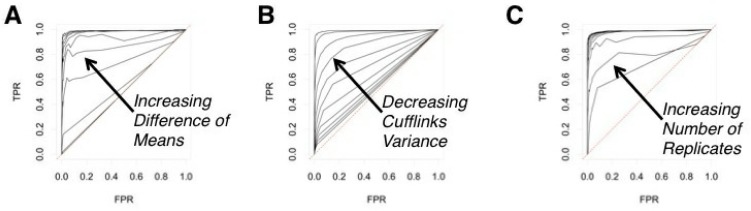
Relative operating characteristic curves (ROC) for the R-EBSeq Method. The false positive rate (FPR) is plotted *vs.* the true positive rate (TPR). Test data sets were generated as described in Methods. Effects of **(A)** the difference of means between two conditions; **(B)** transcript variance; or **(C)** the number of replicates, *M*, on the performance of R-EBSeq.

The following three settings were investigated:

**Mean difference in expression:**
  Fixed: variance—taken from Cufflinks, sample size: 10
  Investigated: influence of difference in expression in 10% of transcripts (“treatment” group). Different levels of differential expression were generated according to the following: 5% of transcripts had *x* mean difference, and the remaining 5% had 2*x* mean difference in expression from the “control” group. The value of *x* was varied from 0, 5, 10,..., 50, and these different values yield ten ROCs visible in Figure 2(A).
**Variance:**
  Fixed: mean difference in expression for DE pattern: 5% of transcripts: 50, 5% of transcripts: 100. Sample size: 10
  Investigated: influence of variance. For the whole “treatment” dataset, we created the ROCs for different additional variance levels. The cufflinks variance was increased by *x* ∈{0, 5,..., 50}, yielding 10 ROCs in Figure 2(B).
**Sample size:**
  Fixed: Mean difference in expression for DE pattern: 5% of transcripts 50, 5% of transcripts 100. Variance: Cufflinks variance
  Investigated: Influence of the sample size. The sample size *n* was investigated at 10 values: *n* ∈ {2, 4,..., 22}, each corresponding to a line in Figure 2(C).

### 2.5. Generating an RNA-Seq Benchmark Dataset for Comparing Various Differential Expression Testing Methods

In order to make comparisons between methods, we needed a benchmark dataset that would provide a common base for all methods. To ensure similarity with experimental conditions, we started with real data published in [13,16]. The control (sam file format) dataset is Tophat processed SRA output [7]. For annotation, we used a GTF file provided by a UCSC database, and to facilitate a direct comparison of various software packages operating on transcript or gene level, we only included in the modified dataset transcripts matching uniquely to particular genes.

The treatment (sam file format) dataset was created by first randomly choosing 100 reads from the control (sam) file. Each of those reads was replicated a number of times corresponding to its order: the first read was copied once, the second twice, and so on, until the 100th was copied 100 times.

To retrieve count datasets for DESeq and BaySeq methods, the created control and treatment files (sam file format) have to be further processed. We used the HTSeq software [17] recommended for DESeq, with default settings. To ensure that all methods will be capable of detecting replicated reads, we have been choosing only reads that had NH:i:1 flag in the sam file, which indicates unique mapping to a particular transcript [18].

The final step of creating the datasets was adding different level of noise to the reads, as described in the following section.

### 2.6. Adding Noise to the RNA-Seq Benchmark Dataset

For the benchmark datasets, to reflect real data, we are adding noise to the RNA-seq data by considering separately reads mapping uniquely to gene, *g*. For each read in this subset, we replicate this read *y_g_* times, where *y_g_* is taken from a negative binomial distribution, that is, *y_g_* *∼* *NB*(*r_g_, p_g_*). The new noisy number of reads for a particular gene, *X_g_*, given that this gene had *N* original reads, is therefore given as:

(10) $$X_{g}=N+Ny_{g}$$

It is well-known that a sum of independent negative binomial random variables is itself negative binomially distributed, with a new *r* parameter that is equal to the sum of the individual *r* parameters. Therefore:

(11) $$Y_{g}\equiv N_{g}y_{g}∼NB\left(N_{g}r_{g},p_{g}\right)$$

Given this, along with the properties of expected values and variances of random variables, we have the following:

(12) $$E(X_{g})=N+\frac{p{\mathrm{Nr}}_{g}}{1-p_{g}}$$

(13) $$\mathrm{Var}(X_{g})=\frac{p_{g}N_{g}r_{g}}{\left(1-p_{g}\right)}$$

It can be shown that when *Var*(*X_g_*) = *E*(*X_g_*):

(14) $$r_{g}={\left(\frac{1-p_{g}}{p_{g}}\right)}^{2}\equiv r_{c};E(X)=\mathrm{Var}(X)=\frac{N_{g}}{p_{g}}$$

Thus, given this negative binomial noise generation model, we can determine the mean and then choose *r_g_* *< r_c_* for underdispersion or *r_g_* *>r_c_* for overdispersion. Because RNA-Seq data are typically overdispersed (*Var*(*X_g_*) *> E*(*X_g_*))[13,14,19], to ensure it, for each gene, *g*, we used the following scheme for obtaining proper *p_g_, r_g_* parameters:

(1) Generate *p_g_* uniformly from distributions *U*[(0.9, 1)].
(2) Use *p_g_* to generate appropriate *r_c_* uniformly, according to distribution *U*[(1.2, 2)].

## 3. Results and Discussion

### 3.1. Gene Count as a Negative Binomial Random Variable

Most of the existing models for RNA-seq analysis employ some form of a parametric distribution of gene counts, typically a gamma-Poisson or a negative binomial, like, e.g., Bioconductor software [20] suites: edgeR, DSS, DESeq or BaySeq). Below, we briefly explain the main reasons for this particular model.

We begin by exploring what an appropriate description of the gene count data may be, and we focus first on the capture step, where members of the library are chosen for sequencing. Let the total number of molecules in a library be *N*, the total number of mapped reads, *n*, and the number of molecules in the library corresponding to the gene, *i*, be *m_i_*. If the capture process is unbiased (we consider capture bias and its variability later), then the probability of capturing a molecule that corresponds to gene *i*, which we denote as the probability of success for gene *i*, is *p_i_* = *m_i_/N*. Because by experimental construction the library size is very large compared to the total number of mapped fragments (*N* ≫ *n*) and only in very exceptional circumstances is an *expressed* biological entity represented very few times in the library (*m_i_* ≫ 1), then for all practical purposes, the probability of success is constant for all capture events. Thus, from the viewpoint of gene *i*, one can cast this RNA-seq experiment as a series of *n* trials, where in each trial, there is some probability of success, *p_i_*. Now, if we denote by *x_i_* the number of reads that map to gene *i*, given the properties of the RNA-seq experiment as outlined above, the probability of gene *i* having *x_i_* reads follows a binomial distribution [21].

Given the large number of genes in any organism, in general, *p_i_* ≪ 1. Since the mean and variance of this aforementioned binomial distribution would be, respectively, *μ* = *Np_i_* and *σ*^2^ = *Np_i_*(1 *− p_i_*) [21], the Fano factor (*σ*^2^*/μ* =(1 *− p_i_*)) would be approximately one for most genes, similar to that predicted by a Poisson distribution (mean equals variance). Therefore, in a situation where the same library is sequenced multiple times with the same total number of mapped reads *n*, a Poisson distribution should be an adequate representation for most gene counts. This was indeed found to be the case for 99.5% of genes from human tissue samples [19].

**Figure 3:**
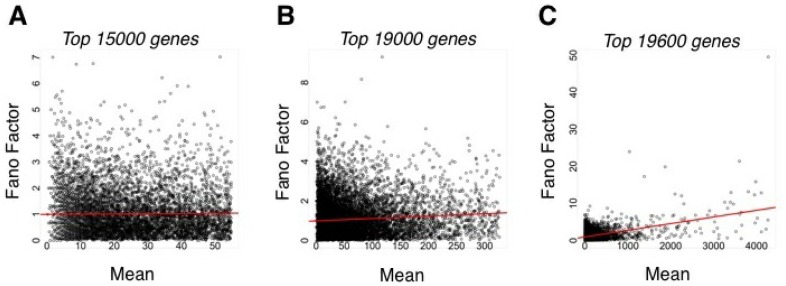
Noise properties of RNA-seq technical replicate data. In every panel, the mean *vs.* the Fano factor for individual genes is plotted. Data were taken from [19]. Black circles correspond to individual genes, and the red line corresponds to a linear regression of the mean *vs.* the Fano factor for the indicated number of genes. Behavior of the **(A)** first **15,000** genes; **(B)** first **19,000** genes; or **(C)** first **19,600** genes as ranked by increasing mean.

This binomial model also suggests that as the mean number of counts, or, analogously, *p_i_*, increases, then the Fano factor should decrease, leading to so-called “underdispersion” relative to a Poisson model. However, technical replicate data from sequencing runs using the same library show that the opposite is true; as the mean number of counts increases, so too does the Fano factor (Figure 3)[19]. This is not evident for the majority of genes with relatively low means (Figure 3(A)), but becomes apparent when higher mean genes are considered (Figure 3(B,C)). Thus, even when one considers only capture variability, a so-called “overdispersion” relative to a Poisson model is evident. Such overdispersion is likely due to bias in the capture process that varies from run to run.

A negative binomial model can adequately capture such overdispersion [13,14], and it describes the probability of needing a particular, but random total number of trials to get a fixed number of failures, given a constant probability of success in each trial. Thus, for a negative binomial model, the experimentally-fixed total number of mapped reads, *N*, has no clear physical interpretation. Therefore, the negative binomial distribution may be thought of as giving an empirical description of RNA-seq count data.

### 3.2. An Empirical Bayes Framework to Detect Differential Expression from RNA-Seq Data

One goal of an RNA-seq experiment is to determine what transcripts (or genes) have significantly different expression levels between various biologically-meaningful conditions. In developing a new method for differential expression analysis, we kept in mind the following three key features of current RNA-seq experiments. First, because count data are not always linearly proportional to the original number of transcripts in the biological sample, converting counts to FPKM measurements for the purposes of differential expression testing is warranted when information sharing across genes is desired, and we wish to remove the size effect. Second, the monetary cost of a single RNA-seq run is high, so having a large number of biological replicates is unlikely. However, differential expression analysis relies on a precise estimate of a transcript’s variance, which is problematic with few replicates and impossible with no replicates. Therefore, information sharing across transcripts to improve variance estimates is necessary. Third, often, one would like to compare more than two biological conditions, so it is preferable to have the ability to do more than pairwise comparisons. Here, we integrate two previously developed software packages, Tophat-Cufflinks [7,9] and EBArrays [10,11], to create an empirical Bayes framework for differential expression analysis of RNA-seq data. We call this hybrid software R-EBSeq, as it incorporates these three key features mentioned above. Since the EBarrays software employs a Bayesian hierarchical model, it may be used with limited (possibly a single) biological replicates. We note that most other methods of differential expression analysis for microarray data, like limma or SAM [20], rely on in-sample variance estimation (limma’s moderated *t*-test) or permutation testing (SAM) and are, therefore, more vulnerable to the small sample size effects.

Before formulating an empirical Bayes approach to differential expression analysis, we must first consider how to convert the raw RNA-seq data into FPKM. The Tophat-Cufflinks pipeline is well-suited for this task, taking into account all the forms of bias and variability shown in Figure 1 with a rigorous, model-based formulation [7,9]. Cufflinks returns an estimated expression value,*Â_t_*, in FPKM for each transcript, *t*, which is given as:

(15) $${\hat{A}}_{\text{t}}=C_{t}{\hat{X}}_{g}{\hat{\gamma }}_{t}$$

where *C_t_* is a normalizing constant for transcript, *t* (which includes the total, genome-wide number of mapped fragments and adjusted transcript length), *X̂_g_* is the number of fragments mapping to gene, *g* (to which transcript *t* belongs), and *γ̂_t_* is the fraction of *X̂_g_* attributable to transcript, *t* (0 *≤ γ̂*_t_ ≤ 1). Cufflinks also returns an estimated confidence interval for *Â_t_*, from which, based on the approximately normal behavior of *A_t_* (see [7] and Methods), the variance of *A_t_*, which we denote as *V*_*A*_*t*__, can be estimated. Thus, by using the Tophat-Cufflinks pipeline, we obtain estimates of the mean and variance of each transcript’s expression level (in FPKM).

A concern of some researchers is that transforming discrete count data into continuous FPKM data results in a loss of information related to the magnitude of the number of counts. To illustrate this concern, consider the case when transcript *a* with length 1 kb has two counts in Condition 1 and four counts in Condition 2, whereas transcript *b* with length 5 kb has 10 counts in Condition 1 and 20 counts in Condition 2. If Condition 1 and Condition 2 have the same number of total, genome-wide mapped reads, then in terms of FPKM, the expression patterns of transcript *a* and *b* are identical. However, on the level of counts, the differential expression of transcript *b* is clearly more significant than that of *a*. This is consistent with the well-known fact that there is a length bias for detecting differential expression in RNA-seq experiments [22,23], which could possibly interfere with the information sharing across transcripts in the EBArrays model. Unfortunately, when converting counts to FPKM, the only way to retain the read length information is to propagate the variance of *X_g_* into the variance of *A_t_*, and Cufflink’s estimate of this variance, *V̂*_*A*_*t*__, indeed does this [7].

A previously developed software package called EBArrays uses a hierarchical, empirical Bayes model to analyze differential expression analysis in microarray studies [10,11]. This model is described in detail in [10,11] and also in the Methods. Conveniently, this software and model address the last two key features mentioned above: information sharing to improve variance estimates and the ability to do more than pairwise comparisons between arbitrary many expression patterns. Thus, by combining the Tophat-Cufflinks pipeline for generating FPKM along with the EBArrays software, we can obtain a hybrid package that is suitable for analysis of RNA-Seq data given the three key features listed above.

The output of the Tophat-Cufflinks pipeline for every condition are estimates of the mean and variance of *A_t_* for each transcript *t* (*Â_t_* and *V̂*_*A*_*t*__), which specify an associated normal distribution. However, these parameters cannot be used as a direct input to EBArrays, as it expects expression values for various conditions. Therefore, to couple these two pieces of software, for each transcript, we generate *M* replicates from a normal distribution with mean, *Â_t_*, and variance, *V̂*_*A*_*t*__, and, then, use these replicates as the input to EBArrays (see Methods). As indicated already above, we call this combined pipeline R-EBSeq.

**Figure 4:**
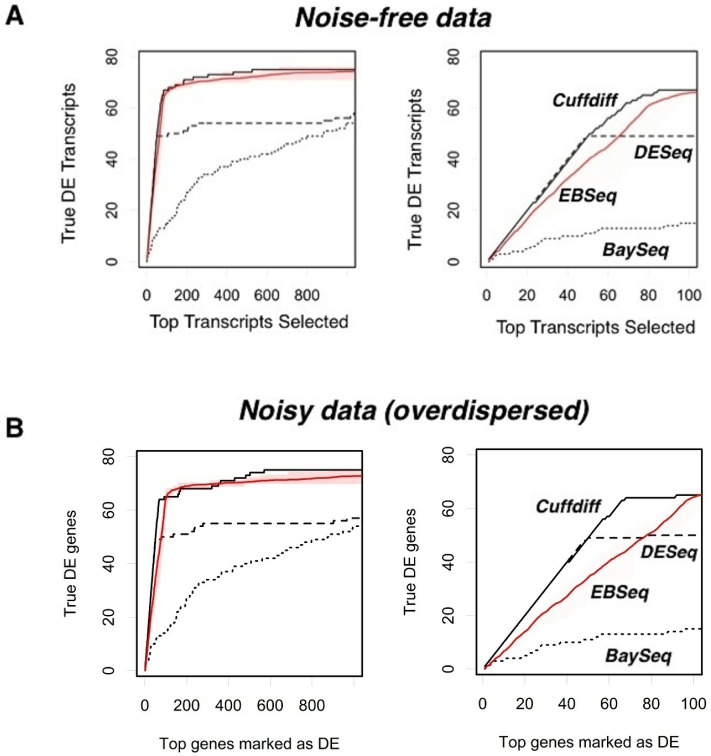
Comparison of true discovery rates for various RNA-Seq differential expression testing methods. The test data sets were generated and various software suites implemented, as described in Methods. Each panel contains two plots; the plot on the right is a zoomed-in version of the plot on the left. On the y-axis is the number of correctly-identified transcripts, and on the x-axis is the number of transcripts selected (in order of increasing *p*-value). DE stands for differentially expressed. In every plot, the thick black line corresponds to Cufflinks, the thick red line to R-EBSeq, the large-dashed black line to DESeq and the small-dashed black line to BaySeq. These lines are also labeled as indicated. The shaded region surrounding the R-EBSeq curve depicts the range of 20 independent runs. **(A)** Performance of the various methods with noise-free data; **(B)** Performance of the various methods with noisy data. Noise was added as described in Methods, and the data are overdispersed, as typical for RNA-seq data.

To evaluate how R-EBSeq performs, we generated a test data set according to the underlying empirical Bayes model, imposed differential expression on a subset of transcripts from this data set and, then, calculated the performance of R-EBSeq in terms of true positive and false positive identifications (see Methods). Such plots of the false positive rate *vs.* the true positive rate are called receiver operator characteristic (ROC) curves. An ROC curve along the *x* = *y* line implies a very poor algorithm that performs no better than random choice, whereas an ROC curve that peaks high above the *x* = *y* line, at low *x* values, implies a very good algorithm. We investigated how three characteristics of a transcript affect the ROC curves: the difference of means between two conditions (Figure 4(A)), the variance of the transcript expression level (Figure 4(B)), and the number of replicates, *M*, used as input to the software (Figure 4(C)). In general, we see that R-EBSeq is capable of very good behavior in terms of the ROC curves. As expected, as we increase the difference of means between two conditions and/or decrease the variance, the ability of R-EBSeq to identify truly differentially expressed genes improves. Increasing the number of replicates, *M*, also improves the performance of R-EBSeq, likely because R-EBSeq is able to get a better estimate of a transcript’s variance.

### 3.3. Comparison of the R-EBSeq Approach to Published Differential Expression Software

There are several packages that currently exist for performing differential expression analysis on RNA-seq data. One method that is also based on FPKM is Cuffdiff, which is part of the Cufflinks pipeline [7]. We also want to consider count-based methods; two highly-used ones are DESeq [13] and BaySeq [14]. To determine how R-EBSeq compares to these three established methods, we need a common benchmark data set where the differentially expressed transcripts are known. However, generation of such a data set is not a trivial task, because using Cuffdiff requires altering a data set on the level of sequencer reads, whereas the input to count-based methods simply require a table of counts. To our knowledge, there is no publicly available benchmark dataset for such purposes. We have therefore modified a previously published RNA-seq dataset on *S. cerevisiae* [16] at the level of reads so as to create a new dataset in which the differentially expressed transcripts are precisely known (Table 1).

**Figure S1:**
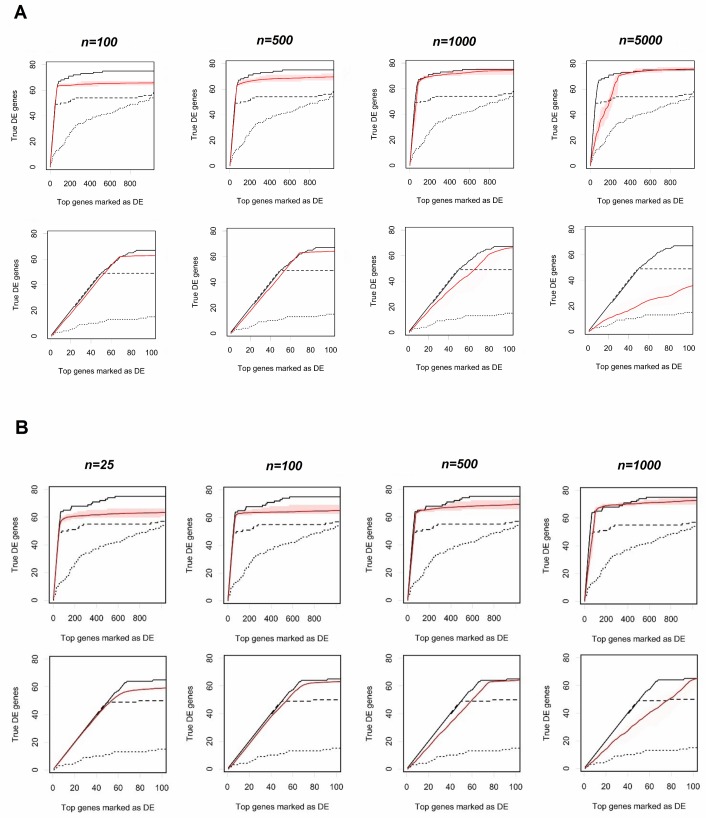
Performance of R-EBSeq Against Other Methods as a Function of th Number of Replicates. In all panels, the thick black line is Cuffdiff, the solid red is R-EBSeq, the large dashed black line is DESeq and the samll dashed black line is Bayseq. The number of replicates for each plot is indicated by the value for *n* above the graph. In each panel, the bottom row (top 100) is zoomed-in version of the top row (top 1,000). **(A)** Performance in the case of noise-free data; **(B)** Performance in the case of overdispersed, noise augmented data.

Before comparing the four software packages, we had to select operating parameters for R-EBSeq. Although above idealized data (*i.e.*, those not coming from sequencing reads), we found that increasing the number of “replicate” (model parameter reflecting our confidence in Cufflinks variance, not to be confused with technical or biological replicates of RNASeq data) used for R-EBSeq always resulted in better performance; with this real data, there is an optimum number of “replicates” (Figure S1). Decreasing the number of replicates allows for better performance in detecting strongly differentially expressed transcripts (at the beginning/left part of the curves) at the expense of detecting weakly differentially expressed transcripts (at the end/right part of the curves). This optimum number depends on the level of noise (compare Figure S1(A) to S1(B)); and in our specific example, it is found to be *≈*1,000 replicates for noise-free data and *≈*100 replicates for noisy data. We reasoned that this behavior is likely due to mismatch between the assumed normal model for FPKM values and the actual distribution, which can be skewed particularly when transcript fractions, *γ_t_*’s, are close to 1 (truncation effects). Most of the investigated differentially expressed transcripts have *γ_t_* =1 (data not shown). Thus, a potential direction for future work with R-EBSeq is to improve the normal distribution assumption for FPKM data. However, changing the normal assumption may slow down the software significantly, since using the normal distribution allows for a clean analytic solution of the posterior distribution in the EBArray model and, therefore, fast computation. It is not clear whether appropriate changes to the FPKM distribution assumption would also be solvable analytically. In a practical setting, we recommend that one selects the number of replicates based on some self-consistency test, like e.g., the cross-validation Shao [15].

Another item to consider before comparison is filtering; that is, throwing away lowly and highly expressed transcripts that are likely to “confuse” EBArrays software, as the differential expression model could become unstable for very low or very high values of *p* and limited biological replicates [11]. Although we could not apply such filtering for Cuffdiff (it is done internally), we could apply filters to R-EBSeq, DESeq and BaySeq. We set these filters to remove 1% of the most lowly expressed transcripts and 0.1% of the most highly expressed transcripts and verified that changing these filter levels only served to degrade performance (data not shown). While these levels are arbitrary and may be dataset-dependent, such a thresholding is a necessary part of differential expression analysis, and future research in this direction is warranted.

**Table 1:** Benchmark dataset for software comparison. The 79 artificially modified transcripts in RNA-seq dataset on *S. cerevisiae* [16] used to generate two artificial datasets (denoted here as Ctrl and Treat) for our software performance comparison study. For each transcript listed in the first column, the specific number of reads listed in the second column is manually added to force differential expression in the Treat group. All the remaining transcripts are left unchanged. Last two columns provide the observed counts as reported by the software.

**Transcript ID**	**Added**	**FPKM-Ctrl**	**FPKM-Treat**	**Counts-Ctrl**	**Counts-Treat**
YHR099W	4	7.67	8.02	42	42
YOR096W	5	7,180.96	7,163.06	1,306	1,306
YPL131W	6	3,665.72	3,658.45	1,645	1,645
YJR091C	10	24.82	28.1	45	45
YDR098C	11	239.1	255.49	86	97
YAL042W	14	150.18	163.53	102	116
YKL160W	20	527.66	613.6	89	109
YGR192C	21	21,270.8	21,176.2	7,392	7,413
YGL076C	22	2,797.82	2,828.35	575	575
YER178W	23	580.38	600.36	348	348
YER136W	24	193.87	214.65	119	143
YAL017W	25	27.83	34.43	67	92
YDL040C	29	77.41	89.84	96	125
YMR315W	35	337.83	379.29	178	196
YHR158C	37	26.66	38.24	48	85
YHR159W	38	12.74	42.8	11	11
YML024W	41	10,985.1	11,140.6	1,191	1,232
YEL034C-A	44	1,638.82	1,756.89	534	578
YBL030C	45	357.3	417.75	140	140
YAR014C	46	33.18	33.03	48	94
YDR037W	47	734.6	762.37	664	664
YOL086C	48	11,798	11,745.5	4,996	5,044
YJR109C	49	82.5	98.29	132	181
YDR448W	51	17.06	65.11	16	42
YDR209C	52	758.86	1,043.42	82	134
YDR105C	53	59.9842	104.932	51	104
YDL130W	54	6,880.78	7,315.38	596	596
YBR191W	55	6,798.73	6,972.36	752	807
YDR083W	55	93.1	122.52	61	116
YBR110W	58	107.46	159.57	66	124
YBL092W	59	10,264	10,540.1	1,530	1,589
YCR012W	61	3,729.34	3,796.95	3,705	3,766
YOR378W	63	6.59	56.45	2	2
YML063W	64	4,559.77	4,657.21	1,545	1,609
YOR224C	65	840.65	1,120.23	119	184
YLR109W	66	7,990.36	7,954.82	1,601	1,667
YFR031C-A	67	2,903.13	3,014.11	531	598
YGR148C	67	3,850.87	4,096.11	450	450
YDL073W	68	36.83	62.37	75	143
YML059C	69	5.8334	20.6487	19	19
YPR089W	70	31.3807	60.7936	58	128
YDR226W	71	1,914.74	2,064.52	544	579
YPL104W	71	23.56	65.08	27	98
YOR122C	72	3,148.49	3,553.19	386	458
YHR102W	73	14.09	14.03	33	106
YDR023W	74	524.92	587.48	409	409
YBL055C	75	97.12	170.68	45	45
YBL091C	76	204.38	277.81	125	125
YJL090C	77	17.48	55.69	25	25
YJL188C	78	5,103.93	5,614.29	802	880
YGR106C	79	728.12	724.89	277	356
YJR072C	79	200.13	285.26	138	138
YOR123C	81	110.42	180.59	93	174
YPL154C	82	398.23	480.465	203	203
YPR074C	83	404.86	449.98	384	384
YFL039C	84	2,343.94	2,427.94	1,093	1,177
YDR050C	85	9,632.29	9,751.83	3,113	3,198
YMR142C	85	4,407.12	4,387.52	1,041	1,126
YDL191W	86	7,255.86	7,223.59	272	272
YGL102C	87	3,688.69	3,785.85	1,686	1,773
YHR193C	88	2,017.73	2,008.75	547	547
YKL145W	89	360.33	436.22	227	316
YMR012W	90	95.84	121.20	191	281
YOR298C-A	92	1,066.95	1,439.52	190	282
YGL012W	93	297.35	375.37	169	169
YDR212W	94	263.12	328.14	219	313
YBR048W	95	3,710.64	4,062	353	448
YBL052C	96	28.92	74.09	47	47
YAL041W	97	50.87	50.65	65	65
YAL038W	98	3,610.93	3,613.65	4,517	4,555
YPL220W	98	5,828.52	6,028.35	312	410
YKL060C	99	17,531.1	17,570.5	9,999	9,999
YPR145W	99	563.698	629.111	526	526
YOR369C	100	18,149.4	18,068.7	3,126	3,226
YER165W	101	418.29	485.05	421	472
YLR359W	101	985.91	1,065.82	664	765
YBR121C	113	402.57	466.61	385	498
YML009C	123	691.233	2,465.9	29	91
YNL178W	146	8,261.72	8,516.73	2,245	2,300

After determining the number of replicates to use for R-EBSeq and the filtering parameters, we proceeded to compare the performance of these four software packages (Figure 4). We first considered the case of noise-free data (Figure 4(A)). Cuffdiff performs the best and is followed closely by R-EBSeq. For the top 100 selected transcripts (right plot), DESeq performs very well for slightly over half of the differentially expressed transcripts, but struggles finding the others. Additional simulations showed that this inability of DESeq is actually due to a problem with the conversion of the read level data to count level data with the software HTSeq, as opposed to that of DESeq itself, in that HTSeq simply failed to convert a subset of transcripts (data not shown). When we eliminated transcripts based on the ability of HTSeq to process them, DESeq performs markedly better (data not shown); however, eliminating transcripts in such a biased manner is undesirable, and we therefore proceeded without this elimination. Importantly, we also tested these software packages with noisy data, ensuring that such noise is overdispersed, as is typical for RNA-seq data (see Methods). Although the noise clearly had an effect on the total number of transcripts that could be identified within the top 1,000, this noise did not change the relative performance of the four methods. Thus, we conclude that Cuffdiff offers the best performance, and R-EBSeq is a very close second. The count-based methods, DESeq and BaySeq, did not perform as well as the FPKM-based methods.

### 3.4. Application of R-EBSeq to Experimental Data-Illustration of Multiple Conditions Comparison

A convenient feature of R-EBSeq that was inherited from EBArrays is the ability to easily specify patterns for multiple condition comparison. We illustrate this feature here using RNA-seq datasets for a DNA damage response with or without *tp53*, a gene that is lost in over 50% of human cancers and is a central player in the DNA damage response (reviewed in [24]). RKO colon carcinoma cells, which are wild-type for p53 protein (WT), or RKO cells where both copies of *tp53* have been knocked-out (KO) were treated with etoposide, a DNA-damaging agent, or DMSO (control) and then assayed for gene expression via RNA-seq [25]. One biological question of interest is to identify which genes change as a function of etoposide without regard to p53-status and which genes are p53-dependent without regard to etoposide-status. It is difficult to identify such genes with traditional pairwise differential expression analysis, particularly because etoposide is used to upregulate p53 expression [26,27,28,29].

To determine potential p53- and etoposide-specific genes, we first specified “patterns”. Pattern specification is the aspect of R-EBSeq (and formerly EBArrays) where one defines how many unique population means we expect and which sample we would expect which population mean to correspond to. In our case, we have four samples and three different conditions we are interested in: p53-specific, etoposide-specific and non-specific (null). To specify these conditions, we consider two different population means, 1 and 2, which are distributed as “patterns” among the conditions according to Table 2. For the pattern etoposide, we require that a gene have a different mean in all etoposide-treated samples. For the pattern, p53, we require that a gene only have a different mean in the WT cells (upon etoposide treatment, which increases p53 levels that are otherwise negligible). For the pattern null, we require that genes have the same mean in all populations. Running R-EBSeq with this setup results in each gene having a posterior probability for each pattern. Thus, the higher the posterior probability, the more likely a gene belongs to a particular pattern.

To analyze the results, we first identified genes having greater than 0.99 posterior probability of belonging to either the etoposide or p53 pattern (Supplemental Table S1). We then compared the high posterior probability gene lists to those in the MSigDB to find those which exhibited significant overlap [30]. The top two hits for the etoposide pattern are *PEREZ_TP53_TARGETS* and *PUJANA_ATM_PCC_NETWORK*. Although it may seem illogical that a list of p53 targets have significant overlap with a list of etoposide-specific genes, this is actually not that surprising and, in fact, expected. There are many proteins that have functional redundancy with p53, so if p53 is not there, it is likely other proteins serve to provide a compensatory effect in the presence of etoposide. Because many p53 targets have been defined as those which respond to DNA damage and many of these targets are likely regulated by compensatory actions, one would expect a large overlap. For the second hit, ATM is a well known DNA-damage related protein, and therefore, its presence is understandably etoposide-related. Some top hits for the p53 pattern are UV response sets (*DACOSTA_UV_RESPONSE_VIA_ERCC3_UP* and *ENK_UV_RESPONSE_KERATINOCYTE_UP*) and stem cell related sets (*NUYTTEN_EZH2_TARGETS_DN* and *BENPORATH_NANOG_TARGETS*). The presence of the UV sets imply that there may be some distinct pathways that are utilized in response to etoposide-mediated DNA damage *vs.* UV-mediated DNA damage and that p53 allows response to both types. The presence of the stem cell sets are in line with current thoughts that p53 plays a large role in stem cell function (reviewed in [31]). Thus, by performing this multiple condition comparison with an appropriately chosen pattern, we were able to find a p53 function related to stem-cells that did not depend on DNA damage.

**Table 2:** Patterns for multiple conditions comparison. Entries correspond to the population mean which sample is assumed to come from, given the pattern indicated. Pattern names are indicated in the row, whereas samples are indicated in the columns. WT: wild-type; KO: knock-out; D: DMSO (control); E: etoposide.

	**WT-D**	**WT-E**	**KO-D**	**KO-E**
Etoposide	1	2	1	2
p53	1	2	1	1
Null	1	1	1	1

## 4. Conclusions

Here we have investigated several aspects of RNA-seq differential expression analysis. In the first part of the paper, we have suggested the empirical Bayes method for RNA-seq data analysis based on FPKM measurements (called R-EBSeq), which combines two previously developed and powerful software suits: Cufflinks and EBarrays. R-EBSeq has two important features. First, it shares information across genes (or transcripts) to get better variances estimates when there are few or no replicates. The use of FPKM makes the sharing straightforward and, unlike in some other empirical Bayes RNA-seq software, does not require additional pre-processing. Second, it is capable of doing multiple conditions comparisons in an easy-to-implement manner, as we demonstrate with experimental data. In the second part of the paper, we created the benchmark dataset for RNA-seq differential expression analysis and used it to compare R-EBSeq to Cuffdiff, DESeq and BaySeq software suits developed for RNA-seq analysis. We find that FPKM-based Cuffdiff gives the best performance, followed closely by R-EBSeq. DESeq and BaySeq, which are count-based methods and do not perform as well. Overall, R-EBSeq seems to offer reasonable performance with the flexibility of multiple comparisons and rigorous treatment of information sharing when there are few replicates.

## Supplementary Files

Supplementary File 1 Supplementary (ZIP, 852 KB)

## Appendix

For the description of the EBarrays LNNMV code, see *nolog.R* file.
